# Edinburgh’s Part in the History of Anæsthesia

**Published:** 1870-02

**Authors:** James Y. Simpson

**Affiliations:** Edinburgh


### Edinburgh's Part in the History of Anæsthesia. An Answer to Dr. Bigelow, of Boston.

BY SIR JAMES Y. SIMPSON, EDINBURGH.

———

Edinburgh, January 3d, 1870.

Dear Sir:—There has been sent to me from America, a Chicago newspaper, containing a letter of yours, which is alleged to have been published in a late number of the Boston "Medical and Surgical Journal." In this letter you speak of the bestowal upon me, some months ago, by my fellow-townsmen, of the rank of an Honorary Burgess of Edinburgh, and comment in terms of bitterness upon the subject, and upon what I said, or rather upon what I did not say, on that occasion.

I feel assured that if you or any one else had felt as nervous and timid as I did on rising to address the public meeting which witnessed the presentation, you would not be astonished at anything I did allude to, or did not allude to; or that I failed in adverting to numerous matters to which I might have adverted.

The gravamen of your charge is this:—

In his extempore address to me, on the occasion in question, the Lord Provost thought fit to allude to some of my professional investigations, and especially to those bearing on Anæsthetics, Acupressure, and Hospitalism. He spoke of the application of *chloroform* to the assuagement of human suffering, as among the "greatest of medical discoveries in modern times." In replying, on the spur of the moment, to these remarks, I stated simply in a sentence the amount to which chloroform was now used for anæsthetic purposes, by adverting to the great extent to which it was manufactured by one single firm at the present day. I might, if there had been time, have added evidence of the extent to which it has superseded all previous anæsthetics, by stating the amount of its manufacture by other firms here and elsewhere. But I had many other subjects to advert to besides chloroform, and only a few short minutes within which it was expected to include them all. According, however, to your views, I am very deeply blameable for not taking up a subject which the Lord Provost did not allude to, namely, the history of anæsthesia. You hold that I should have entered, to a greater or less extent, into some historical notice of anæsthetic agents. The history of them has always taken me a full hour in my University Lectures, and in these lectures I have year after year heartily paid every due compliment to the most important part borne in the consummation of the practical application of anæsthetics, by America, particularly by the cities of Hartford and Boston, and especially by the energy and genius of Dr. Morton. Surely, however, it would have been sadly out of place on such an occasion, and with such an audience, to have shown that before I discovered the application of chloroform to anæsthetic purposes, numerous other agents had been previously suggested and used for the same object,—as sulphuric ether by Drs. Jackson, Morton and Marcy; as carbonic acid by Dr. Hickman, in imitation of the experiments performed for ages on the poor dogs at the Grotto del Carno; and as nitrous oxide,—an agent extensively employed as a dentists' anæsthetic at the present hour, and which was first proposed some seventy years ago, for "destroying physical pains" during "surgical operations," by Sir Humphrey Davy; or should I, in your opinion, have even gone still farther back in therapeutic history, and described what, doubtless, as a former lecturer you are well acquainted with, namely, the other soporific vapors and measures employed by different olden surgeons, in Greek, Rome and Mediæval times, with the view of rendering their operations painless to the patient? In that way I might have easily shown that the idea of making a patient anæsthetic, before subjecting his body to the knife or cautery, was a kind of knowledge familiar even to non-professionsl writers of mediaeval and of later times, and that some theological authors, like Origen for example, in the third century, allude to the artificial production of anæsthesia in surgery, as a well-known practice; while in reference to Scotland, I might have cited Abbott Bower, who lived and wrote about the year 1400, within ten miles of Edinburgh, as telling us by what means anaesthetic surgery was accustomed to be effected in those days, and what they gave to patients. "*Secandi ut possent sine dolore secari;*" or I might have adduced the Monk Joceline, as alluding with circumstantial details to an alleged instance of it, in the Hagiology of Scotland, as early as the sixth century,—all this and much more might have been mentioned; but all this would have been in my opinion, though not apparently in your opinion, totally misplaced, and greviously out of order, as much as a disquisition on the previous means of arresting surgical hemorrhage in wounds by ligature, torsion, etc., would have been when I adverted for a moment to the subject of acupressure.

In the way of a climax, you terminate one of the paragraphs in your letter with the statement that I was not the "first man" to inhale a vapor to such an extent as to destroy sensibility. Most certainly I was not; and certainly I never was so intensely foolish as to claim to be so. In the course of my investigations I have, however, experimented upon myself with various vapors, the innocuous or the poisonous effects of which upon the economy were previously altogether unknown and unascertained, and I have sometimes suffered in consequence. As a Professor of Therapeutics, you must surely be well aware that the first experiment of breathing a vapor to such an extent as to destroy sensibility was made neither in America nor in our own days. Without adverting to the acknowledged fact that it was accomplished with the vapors driven off from hypnotic vegetable extracts, by the older surgeons, from Hugo de Lucca and Theodoric downwards, let me remind you that Sir Humphrey Davy boldly (and notwithstanding that he had witnessed occasional deaths in animals from it) made the experiment to which you advert, many times upon himself, in the last year of the last century, with nitrous oxide, and found that headache and other pains disappeared under its influence.

About forty years ago, Faraday in this country, and Godman in America, showed, as the result of their observation and experience, that the effects of the inhalation of the vapor of sulphuric ether were quite similar on the nervous system to those produced by the inhalation of the vapor of nitrious oxide gas,—a truth subsequently proved by many pupils in many chemical and other schools, in your country as well as in mine, by their inhalation of ether. Your remarks, as far as I understand them, imply that it is your belief that Dr. Morton was "the first man" of "sufficient courage" to breathe "a vapor" so as to produce a state of anæsthesia. But you must know as well as I do, from the official documents laid before the Senate of the United States, that this is doubtful as regards the course of matters even in America. For it appears in these documents, (1.) That Dr. Jackson avers that he breathed for this object sulphuric ether earlier than Dr. Morton; (2.) That before Dr. Morton made the same experiment upon himself, in 1846, he made it first upon others, and particularly upon his pupil, Mr. Spiers; and (3.) That two years previously (or in 1844), Dr. Marcy, of Hartford, had successfully excised a tumor from a man who had been rendered ansæthetic for the purpose by the vapor of sulphuric ether, whilst at the same early date, in the same city, Dr. Horace Wells had extracted teeth from a dozen or more patients rendered insensible by inhaling nitrous oxide according to Davy's suggestion.

There has lately been raised, I am told, in the city of Boston, a monument in commemoration of the employment of anæsthesia in surgery in that city in 1846. But have the erectors of this monument cut upon it the names of either of your fellow-citizens, Dr. Morton or Dr. Jackson, as the first investigators, or the names of Warren and Hayward, as the first Boston hospital surgeons who operated upon patients under the influence of sulphuric ether? or have they generously inscribed upon its sides any allusion to the fact that two years previously anæsthetics had been inhaled successfully in dentistry and surgery in the neighboring city of Hartford? I have been assured, though it is scarcely credible, that there does not appear upon the monument the name of a single American chemist, dentist, or surgeon. Why is it so? You have the monument. Have you not had the men?

You commence the concluding paragraph of your letter by averring that anæsthetic inhalation "began" (to use your own words) "in this country" (America), "and was first used in the extraction of teeth, and afterwards in capital operations in the Mass. General Hospital, and in obstetrical practice." Your words so far affirm that anaesthetic inhalation, besides being first employed in America in dentistry and surgery, was in your country also "first used" in "obstetrical practice." You must excuse my saying that this last assertion is unaccountably incorrect. The use of anaesthetic inhalation in obstetrical practice was begun and extensively followed out in Edinburgh, weeks or even months before it was tried in Boston, or America. The first case of midwifery in which sulphuric ether was adopted as an anaesthetic occurred here under my care on the 19th January, 1847. On the 1st March, 1847, was published by me, in the "Edinburgh Medical Journal," an essay on the subject, containing a series of obstetrical cases, and a longish discussion of the question of the applicability of anæsthetics to midwifery. It was not, however, according to the published evidence of your townsman, Dr. Channing, till April 7th, that the first case of the employment of anæsthetics in midwifery occurred in America, and the second did not take place till 5th May. (See Dr. Channing's "Treatise on Etherization in Childbirth," p. 26.) But before the date of these two cases the practice had been fully established in Edinburgh and elsewhere. Perhaps you and I, as parties implicated, are not adequate judges as to whether your statement on this point is candid and creditable, or utterly the reverse; but I willingly leave the decision of this to the feelings and verdict of an honorable profession. You think that I am greatly blameable because, in the way of omission, I did not advert to the previous application of sulphuric ether in America as an anæsthetic, when the employment of chloroform was referred to. I think, on the contrary, that you are infinitely more blameable than I am, because, without the slightest reason or ground, and in the way, not of omission, but of deliberate commission, you have, in this letter of yours, attempted to appropriate for your city and country, what indubitably belongs to my city and country, namely, the credit of the first introduction and establishment of anæsthetic inhalation in obstetrical practice.

I have the honor to be,

Yours truly,

J. Y. SIMPSON.

To Dr. Jacob Bigelow, Boston.

*Gynæcological Journal.*

